# Bridging Knowledge and Data Gaps in Odonata Rarity: A South Korean Case Study Using Multispecies Occupancy Models and the Rabinowitz Framework

**DOI:** 10.3390/insects15110887

**Published:** 2024-11-13

**Authors:** Sungsoo Yoon, Wanmo Kang

**Affiliations:** 1 Department of Forest Resources, Graduate School of Kookmin University, 77 Jeongneung-ro, Seongbuk-gu, Seoul 02707, Republic of Korea; yssfran@nie.re.kr; 2 Ecological Information Team, National Institute of Ecology, 1210 Geumgang-ro, Seocheon-gun 33657, Republic of Korea; 3 Department of Forest Environment and Systems, College of Science and Technology, Kookmin University, 77 Jeongneung-ro, Seongbuk-gu, Seoul 02707, Republic of Korea

**Keywords:** Odonata, species rarity, occupancy modeling, Rabinowitz rarity framework, knowledge–data discrepancy

## Abstract

Accurate assessment of species rarity and conservation status requires an approach that integrates data-driven models with established ecological knowledge. In this study, we applied multispecies occupancy (MSO) and latent factor multispecies occupancy (LFMSO) models to estimate the occurrence of 133 Odonata species in South Korea. Using the model outputs, we implemented the Rabinowitz rarity framework to conduct data-based rarity assessments, which were then compared with known ecological information, including geographic ranges, habitat preferences, regional Red List statuses, and citizen science observations. Our findings reveal both alignments and discrepancies between these data-driven rarity assessments and traditional ecological knowledge. For example, species classified as near threatened (NT) or vulnerable (VU) on the regional Red List generally corresponded with high-rarity classifications based on the Rabinowitz framework. However, significant inconsistencies were identified, particularly for certain lentic Odonata species traditionally considered common. These results suggest that spatial biases in field surveys, combined with limited access to data on legally protected species, can impede accurate rarity assessments. These findings underscore the need for standardized survey protocols and improved data-sharing policies for sensitive species to reduce biases and enhance the reliability of rarity assessments. This is essential for effective conservation planning and biodiversity management in freshwater ecosystems.

## Simple Summary

The members of the order Odonata, commonly known as dragonflies and damselflies, play an essential role in freshwater ecosystems. However, identifying their rarity and conservation status is often difficult due to gaps between available data and existing knowledge. In this study, we employed the Rabinowitz rarity classification framework, using outputs from multispecies occupancy models that predict the occurrence of Odonata species in South Korea. We compared the results of these models with established information, such as geographic range, habitat preference, conservation status, and citizen science records. Our findings reveal that species with high need for conservation measures were typically identified as rare or data-deficient. However, notable discrepancies emerged, particularly for species traditionally regarded as common, often inhabiting lentic habitats. This highlights the necessity of standardized survey methods and improved access to data on legally protected species for accurate rarity assessments. Our study emphasizes the importance of enhancing survey protocols and data-sharing practices to provide more reliable species rarity evaluations and support effective conservation strategies for freshwater ecosystems.

## 1. Introduction

Odonata (dragonflies and damselflies) play vital roles as predators in both freshwater and terrestrial ecosystems by regulating the populations of their prey, while also serving as a food source for larger predators such as birds and fish [1,2,3]. Due to their high sensitivity to environmental conditions, including habitat degradation, water pollution, and climate change, Odonata species are considered effective bioindicators of both aquatic and adjacent terrestrial ecosystems [4,5,6,7,8]. Given their ecological significance, numerous rare and endangered Odonata species have become key targets for conservation and restoration efforts [9,10,11,12]. Understanding the distribution and conservation status but also ecological requirements of Odonata species is, therefore, critical for monitoring biodiversity, ecosystem functions, and environmental changes in their habitats.

Current conservation strategies for Odonata typically adopt one of two main approaches: the species-specific approach, which focuses on identifying suitable environments for the survival of conservation-priority species with specific habitat requirements [13,14], and the community-level approach, which examines changes in species composition and richness along environmental gradients on a regional scale [15,16,17]. For example, recent studies in South Korea have largely focused on the habitat needs of two endangered species, *Libellula angelina* and *Nannophya koreana*, as well as the distribution patterns of *Ischnura senegalensis* and *Brachydiplax chalybea flavovittata*, species that thrive in the warmer southern regions of South Korea and are expected to expand their habitats due to global warming [18,19,20,21]. Additionally, shifts in Odonata species composition over time and across environmental gradients have been investigated, often as a part of benthic macroinvertebrate assemblages [22,23,24]. However, as in many other countries, comprehensive studies using ecological models to assess the overall status of Odonata species nationwide have not existed. While the regional International Union for Conservation of Nature (IUCN) Red List [25] currently designates 12 Odonata species as either ‘Vulnerable’ or ‘Near-Threatened’ in South Korea, out of 116 species assessed, the conservation status of many species remains unclear. This is especially true for the 33 species excluded from the regional Red List assessment, primarily due to data deficiencies and uncertain population status. Furthermore, integrating occurrence data from various field surveys into ecological models is essential to overcome data gaps for rare species and to accurately estimate key metrics, such as the area of occupancy (AOO) and extent of occurrence (EOO), both of which are critical for determining Red List criteria.

Species distribution models (SDMs) are widely used to predict the areas occupied by target Odonata species [26,27,28]. However, many studies using SDMs, particularly those based on the Maxent model, rely on presence-only data from opportunistic surveys, which may introduce spatial sampling bias [1,29,30,31,32]. A more challenging issue arises when modeling the distribution of rare or endangered species, as presence data for these species are often under-recorded due to their low detectability [33,34], leading to problems such as model overfitting or underestimation of their actual range [35,36]. Recently, multispecies occupancy models, which account for detection probability using both detection and non-detection (presence–absence) data, have been employed to address these challenges, reducing spatial sampling bias and mitigating imperfect detections to provide more accurate estimates of species distributions [37,38]. Therefore, a data-driven approach using MSO models provides a promising means to enhance conservation strategies, supporting IUCN Red List assessments that still rely heavily on expert opinion [33].

Expert knowledge often diverges from field data, particularly when assessing the extinction or rarity of species. The subjective nature of declaring a species extinct within the IUCN Red List framework highlights how experts weigh various factors differently, based on individual knowledge and experience, which can lead to variability in extinction assessments [39]. This reliance on expert judgment can result in discrepancies, as seen in instances where species believed to be extinct were later rediscovered, often due to gaps in data collection over time [40]. The challenge is further complicated by the limited or restricted accessibility of data on rare or endangered species [41,42], which can lead to incomplete or biased assessments. Bridging the gap between expert knowledge and empirical data is essential for ensuring accurate and equitable assessments of species’ conservation status.

This study sought to address the gap between knowledge and data when assessing the rarity of Odonata species in South Korea by applying MSO models in conjunction with Rabinowitz’s rarity framework. Rabinowitz’s framework has been employed to classify species into eight rarity groups by estimating and comparing their geographic range (wide vs. narrow), habitat specificity (broad vs. restricted), and local population size (large vs. small) based on presence-only records [11,43]. However, relying solely on presence-only records cannot account for spatial sampling biases or the underestimation of species with low detectability. To address these issues, we incorporated the estimated occupied areas for each Odonata species, calculated through MSO models, into Rabinowitz’s rarity framework. This approach enabled the assessment of the rarity and data status of each Odonata species and validated the results against existing knowledge, including species traits, observation data, and current IUCN Red List statuses. Finally, our study explored how our approach can contribute to more informed conservation strategies and practices for Odonata species.

## 2. Materials and Methods

### 2.1. Study Area

**Figure 1:**
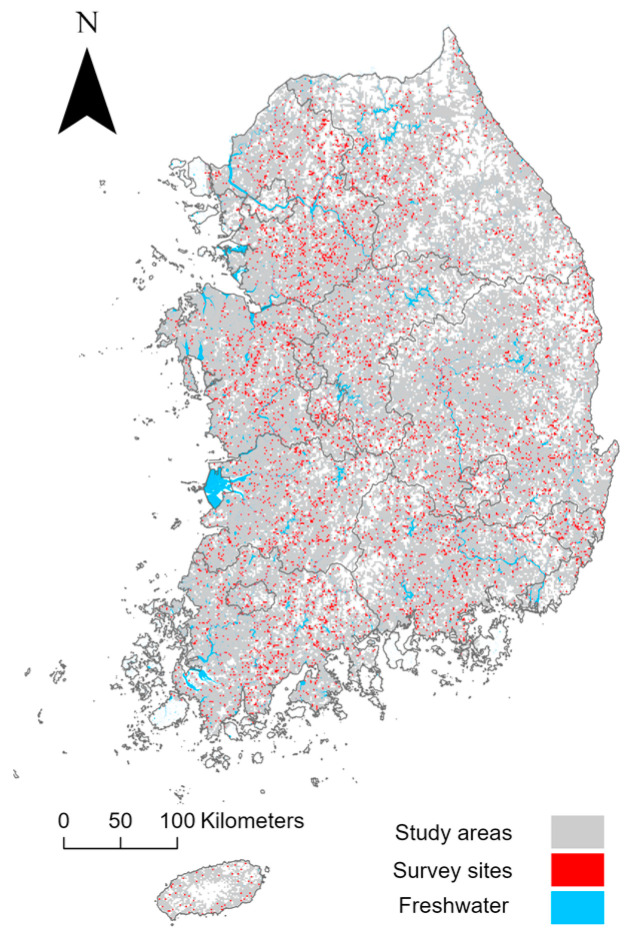
Map of the study area showing survey sites where Odonata species were detected, based on data from the National Ecosystem Survey (NES) and the Survey and Evaluation of Aquatic Ecosystem Health (SEAEH).

This study focused on nationwide gridded areas within the borders of South Korea, East Asia (latitude 33° to 39° N, longitude 124° to 130° E), using a 1 km spatial scale (Figure 1). Island regions were excluded from the study, with the exception of Jeju Island, the largest island located in the southernmost part of South Korea. Additionally, grid cells not intersecting with freshwater ecosystems were omitted. The defined study area covers approximately 77,000 km^2^ and is characterized primarily by riverine ecosystems associated with four major rivers: the Han, Nakdong, Geum, and Yeongsan. In the eastern regions, valleys have developed along the Baekdudaegan mountain range, which runs longitudinally across South Korea. In contrast, the western regions are generally composed of mixed terrains, including agricultural and urban areas in the lowlands and hilly regions. The study area experiences four distinct seasons: a dry and clear spring (March–May) and autumn (September–November); a cold and dry winter (December–February); and a hot and humid summer (June–August). Average summer temperatures range from 19.7 °C to 26.7 °C, while winter temperatures range from 6.9 °C to −3.6 °C. Annual precipitation is approximately 1200 mm, with the majority occurring during the summer months [44].

### 2.2. Data Collection

We used two datasets established from the Survey and Evaluation of Aquatic Ecosystem Health (SEAEH) [45] and the National Ecosystem Survey (NES) [46], chosen for their nationwide coverage across project units and the consistent application of standardized protocols at each survey plot. These datasets provided detection data with replicates from survey sites, meeting the necessary data structure for occupancy models. Both datasets cover the period from 2011 to 2022. We used macrobenthic invertebrate survey data from the 3rd (2011–2013), 4th (2014–2018), and 5th (2019–2022) NES programs, each conducted nationwide over a five-year period and involving taxonomic experts to collect and identify species presence data. The macrobenthic invertebrate surveys from NES targeted key lentic and lotic habitats that represent biodiversity within each of South Korea’s 850 catchment units. The NES employed qualitative sampling at least twice annually (once between March and May, and again between August and October) using D-frame nets, Surber nets, or dredge samplers with mesh sizes smaller than 1 mm. The SEAEH program, which covers streams and estuaries across the nation every three years, also collects benthic macroinvertebrates twice a year at designated sites. For SEAEH, at least three representative sampling plots were selected per site, primarily focusing on riffles. Surber nets were used for plots with depths of 50 cm or less, while dredge samplers were employed for deeper plots.

In addition to the aforementioned surveys, 26 predictors, including elevation, bioclimatic variables, and land-cover data, were gathered based on previous studies [28,47,48,49,50,51,52,53,54,55,56,57,58] (Table 1). Elevation data were obtained from the Advanced Land Observing Satellite (ALOS) Global Digital Surface Model (DSM): 30 m dataset (version 3.2) via the Google Earth Engine platform (https://code.earthengine.google.com, accessed on 10 April 2024). Five land cover-related datasets, representing forest, agricultural, urban, lotic, and lentic areas at a 1 m spatial resolution for 2021, were sourced from the Environmental Geographic Information Service (https://egis.me.go.kr/, accessed on 10 April 2024). Historical bioclimatic data (19 variables) at a 1 km resolution, representing conditions from 1970 to 2000, were obtained from WorldClim (https://www.worldclim.org, accessed on 10 April 2024). Elevation data were preprocessed by upscaling to a 1 km resolution grid using the bilinear resampling technique in ArcGIS Pro 3.2 for further analysis [59].

To validate the Odonata species’ rarity and data status assessed in this study, we collected regional IUCN Red List data, as well as species traits and citizen-science-based observation data [25], for the 133 target Odonata species recorded in the South Korean Odonata checklist. The IUCN Red List categories used included: (1) least concern (LC): species that are currently widespread and abundant; (2) near threatened (NT): species that are close to being threatened and may become so in the near future; (3) vulnerable (VU): species facing a high risk of extinction; (4) data deficient (DD): species for which there is inadequate information to assess the risk of extinction; and (5) not evaluated (NE): species that have not yet been reviewed sufficiently for Red List assessment. For trait data, we focused on two key aspects: (1) geographic range within South Korea, and (2) habitat preferences. Geographic ranges were categorized into six types: nationwide (NW), north (No), mid-north (MiNo), south (So), mid-south (MiSo), and unknown (NA). The distribution of Odonata species was recorded separately based on data from two time periods (2007 and 2021) [60,61], allowing for comparisons of changes in geographic range over time. Habitat preferences were classified as running water (R), still water (S), both freshwater environments (SR), or unknown (NA). Finally, citizen science observations of Odonata species, recorded since 2014, were retrieved from the Naturing platform (https://naturing.net, accessed on 17 August 2024), a well-known citizen science platform for nature observation in South Korea.

### 2.3. Building Occupancy Models

To assess the occurrence of Odonata species across South Korea, we applied both standard multispecies occupancy (MSO) and latent factor occupancy LFMSO models, utilizing data from the NES and SEAEH programs, respectively. In the MSO model, the true presence ($z_{i,j}=1$) or absence ($z_{i,j}=0$) of species i at site j is modeled as a Bernoulli process, as follows:

(1) $$z_{i,j}\sim Bernoulli\left(\psi_{i,j}\right),\;\mathrm{logit}\left(\psi_{i,j}\right)=X_{j}\beta_{i}$$

where the probability of occurrence ($\psi_{i,j}$) is calculated using a logit-link function, which integrates a linear combination of z-score-normalized site-specific covariates ($X_{j}$), including mean annual temperature (bio1), mean diurnal temperature range (bio2), precipitation seasonality (bio15), and the areas of still water (lentic) and running water (lotic) habitats, alongside species-specific regression coefficients ($\beta_{i}$). The site covariates were selected based on a review of 13 previous studies [28,47,48,49,50,51,52,53,54,55,56,57,58] focused on Odonata species’ ecological niche modeling. Only covariates used in at least four of these studies and that showed no multicollinearity (Pearson’s correlation coefficient > 0.75) were included in the final set. The $\beta_{i}$ were treated as random effects drawn from a community-level normal distribution:

(2) $$\beta_{i}\sim Normal\left(\mu_{\beta },{Τ}_{\beta }\right)$$

where $\mu_{\beta }$ represents the average effect of each predictor across the Odonata community, and ${Τ}_{\beta }$ is the variance of these effects among different species. To account for imperfect detection, the observed detection ($y_{i,j,k}=1)$ or non-detection ($y_{i,j,k}=0)$ of species i at site j during replicate k was modeled as a Bernoulli process, conditional on the underlying true presence or absence:

(3) $$y_{i,j,k}\sim \;Bernoulli\left(p_{i,j,k}z_{i,j}\right),\;\mathrm{logit}\left(p_{i,j,k}\right)=\alpha_{0}+\alpha_{1}v_{i,j,k}$$

where the detection probability ($p_{i,j,k}$) is modeled as a function of replicate-specific covariates ($v_{i,j,k}$) along with a species-specific detection coefficient ($\alpha_{i}$). In this model, the number of site visits during replicate k is used as a detection covariate, serving as a surrogate for sampling effort. Similar to $\beta_{i}$, $\alpha_{i}$ is assumed to follow a community-level normal distribution, represented as follows:

(4) $$\alpha_{i}\sim Normal\left(\mu_{\alpha },\;{Τ}_{\alpha }\right)$$

where $\mu_{\alpha }$ is a vector representing the mean effects of each detection covariate across the Odonata community, while ${Τ}_{\alpha }$ captures the variability of these effects among different species. The LFMSO model extends the MSO model by incorporating latent factors to account for residual correlations between species. In the LFMSO model, $\psi_{i,j}$ is further influenced by a term that represents unobserved latent factors, as follows:

(5) $$\mathrm{logit}\left(\psi_{i,j}\right)=X_{j}\beta_{i}+w_{i,j}^{*},\;w_{i,j}^{*}=\lambda_{i}w_{j}$$

The latent process ($w_{i,j}^{*}$) is decomposed into a linear combination of q latent factors and their associated species-specific loadings ($\lambda_{i}$), which indicate how strongly species i responds to each latent factor ($w_{j}$). Specifically, $\lambda_{i}$ is a row vector corresponding to species i from a matrix Λ with dimensions N×q, where N is the number of Odonata species, and $w_{j}$ represents the actual latent factors at site j. This approach efficiently captures the correlations among species, especially when q is much smaller than N. For this study, we initially set q=3 to account for the habitat preference of Odonata species based on their known habitat types (S, R, and SR). However, after conducting initial runs of the LFMSO models, we observed that only one latent factor provided meaningful loading values (constant positive and negative trends for each species within a 95% confidence interval) for the Odonata community, leading us to reduce q to 1.

Before building the occupancy models, we transformed the presence-only data collected from the NES and SEAEH programs into detection–non-detection data aligned with 1 km spatial-resolution grids. The replicates were defined based on the years in which each data source conducted nationwide surveys. The transformed data included 41 species across 253 sites with three replicates from the NES program and 64 species across 2510 sites with four replicates from the SEAEH program. To ensure the efficiency and reliability of the models, we excluded species with fewer than three detection records and sites where no Odonata species were detected across all replicates. However, we retained sites with no Odonata species records if the total number of site visits exceeded the upper 95% threshold, as these sites can provide information on very low habitat suitability and the absence of Odonata species. A Bayesian approach, commonly adopted for fitting complex hierarchical models [38,62,63], was used to estimate parameters in the LFMSO and MSO models. Specifically, the occupancy models were fitted using a Markov-chain Monte Carlo (MCMC) method with the Gibbs sampler algorithm [62], ensuring the convergence of the MCMC chain. All models were run with the following settings: three chains, each with 60,000 samples, 30,000 burn-in samples, and a thinning interval of 30, resulting in 3000 MCMC samples. We applied normal priors to the mean parameters and inverse-Gamma distributions for the variance parameters, along with initial model hyperparameter values suggested by previous studies [62,64]. Model convergence was assessed by confirming that the Gelman–Rubin diagnostic (Rhat) was less than 1.1 and that the effective sample size exceeded 100. If an occupancy model did not converge with the initial settings, we doubled the total number of samples and burn-in samples, as well as the thinning interval value per chain. The final models for each data source were selected based on a Bayesian *p*-value between 0.05 and 0.95, indicating a good model fit when grouping the data by individual sites, as well as the lowest value of the widely applicable information criteria (WAIC) for each data source. The LFMSO and MSO models from each data source were implemented using the ‘spOccupancy’ package [62] in R (version 4.3.3) [64]. Shapiro–Wilk tests (*p* < 0.05) for normality and Spearman’s correlation analysis of the final model estimates were also conducted in R (version 4.3.3) [65] to validate assumptions and assess correlations. For individual Odonata species, sites with a predicted probability of occurrence greater than 0.5 were considered occupied areas for applying the Rabinowitz rarity framework. Additionally, for further discussion on conservation strategies, we estimated Odonata species richness based on the NES and SEAEH data by summing the latent occurrence states predicted by the final models.

### 2.4. The Rabinowitz Rarity Framework and Results Validation

The geographic range, local population size, and habitat specificity were calculated based on the occupied sites (1 km^2^ grids) for each Odonata species to classify two rarity sub-groups for each attribute within the Rabinowitz rarity framework. The geographic range corresponds to the EOO concept in the IUCN Red List assessment. We calculated the geographic range of individual Odonata species by creating a minimum convex polygon (MCP) that encompasses all the occupied grids. The median area (in km^2^) of the grids that intersect with the MCPs of each Odonata species was used to classify the species into ‘wide’ and ‘narrow’ range groups. For species with two or fewer occupied sites, the geographic range was calculated as the total area of the occupied grids. The local population size is represented by the AOO, which was calculated as the number of occupied grids for each Odonata species. Similar to the geographic range, species were categorized into ‘large’ and ‘small’ population groups based on the median value of AOO.

**Figure 2:**
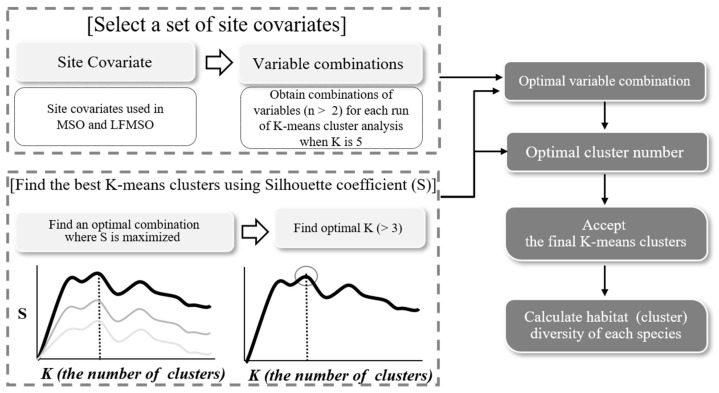
Workflow for determining the optimal site covariates and habitat clusters for Odonata species in South Korea using k-means clustering.

Habitat specificity was assessed using the site covariates employed in the occupancy model to determine the optimal number of habitat types and sites in South Korea through k-means clustering (Figure 2). In this process, the optimal combination of site covariates identified for habitat clusters included bio1 (mean annual temperature), lentic (still water habitats), and lotic (running water habitats). The silhouette coefficient, which measures the quality of clustering, was maximized (silhouette coefficient = 0.43) when using these three variables and setting the number of clusters K to 7. This indicates that this combination best represents distinct 7 habitat types across the study area. The habitat specificity of species i ($HS_{i}$), based on the Shannon diversity index [66], was then calculated for each species as follows:

(6) $$HS_{i}=-\overset{n}{\underset{hc=1}{\sum }}p_{hc}lnp_{hc}$$

where $p_{hc}$ denotes the proportional abundance of a particular habitat cluster assigned to species i. Odonata species were classified into ‘broad’ and ‘restricted’ habitat groups based on the median value of $HS_{i}$ among all Odonata species The clustering of habitat types was conducted using the kmeans function, with silhouette coefficients calculated using the silhouette function of the cluster package [67], while additional calculations for habitat specificity were performed with base functions in R (version 4.3.3) [65].

The eight rarity classes were derived from the Rabinowitz framework, which considers three attributes, each divided into two groups. We visualized the relationships between rarity classes and the current regional IUCN Red List, known geographic ranges, habitat types, and citizen-science-based observation data using bar graphs and box plots. Species excluded from the final rarity classifications were categorized into two groups: (1) not observed (NO), which includes species with no records in the NES and SEAEH data; and (2) data deficient (DD), which comprises Odonata species with detection records that were either insufficient for occupancy modeling or showed an occurrence probability of less than 0.5 across all sites. Differences in the number of observations retrieved from the citizen science platform Naturing.net among rarity and data status groups were assessed using the Kruskal–Wallis test followed by Wilcoxon rank-sum pairwise comparisons with Bonferroni corrections. The Shapiro–Wilk test for normality indicated that the data did not meet the assumption of normality (*p* < 0.05), leading us to select non-parametric methods. The Shapiro–Wilk test was conducted using the shapiro.test function, the Kruskal–Wallis test was performed using the kruskal.test function, and Bonferroni-adjusted pairwise comparisons were carried out with the pairwise.wilcox.test function in R (version 4.3.3) [65]. Additionally, we discussed the possible reasons for the low data availability for NO and DD Odonata species, referencing the ecology of Odonata species in South Korea (Table S1), along with a review of the data sources and the analytical framework used in this study.

## 3. Results

### 3.1. Occupancy Model Results

**Figure 3:**
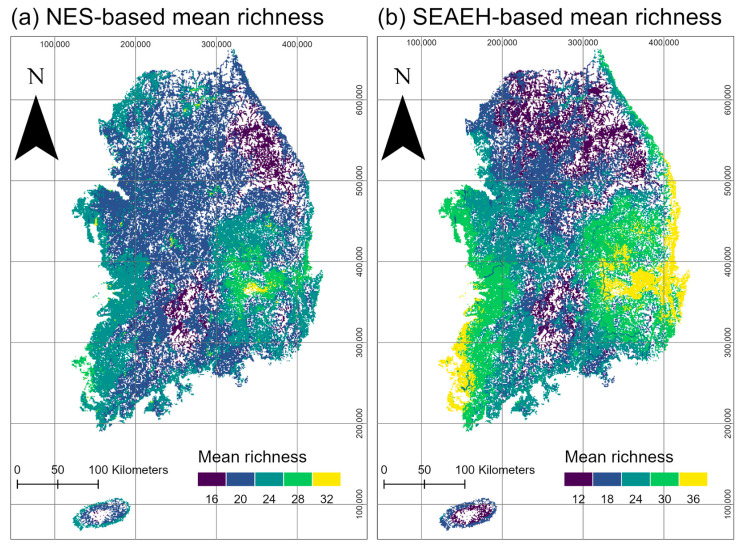
Mean species richness maps predicted by LFMSO models using data collected from two nationwide surveys, (**a**) NES and (**b**) SEAEH, in South Korea.

Regardless of the data sources, the LFMSO models were chosen as the final models due to their lower WAIC values (LFMSO vs. MSO: 8495 vs. 8737 for the NES data and 71,436 vs. 83,355 for the SEAEH data). The general trends in the estimates of site covariates were largely consistent between the LFMSO models based on NES and SEAEH data, with the exception of bio15, which exhibited an opposing effect on the occurrence probability of the Odonata community (Table 2). Among the site covariates, bio1 and bio12 demonstrated stronger impacts on occurrence probabilities compared to the others (Table 2 and Table S2). Lentic and lotic environments had similar magnitudes of impact on occurrence probability, although they acted in opposite directions. Additionally, the number of visits to study sites positively influenced detection probabilities at both the community (Table 3) and species levels (Table S2). The mean richness predicted by both LFMSO models indicated areas of high species richness, particularly near the downstream sections of large rivers in lowland regions (Figure 3a,b). Conversely, lower predicted mean richness values were observed in mountainous terrains, primarily in the eastern and mid-southern regions of South Korea.

### 3.2. Rarity of Odonata Species and Results Validation

Among the 133 target species, only 62 species could be classified into rarity classes based on the Rabinowitz framework. This limitation was primarily due to low data availability for occupancy modeling, as more than half of the target Odonata species lacked sufficient data (Table 4). Specifically, 35 species were categorized as NO, whereas 36 species were designated as DD, accounting for 26.3% and 27.1% of the total species, respectively. The rarest group (NRS) comprises 16 species, representing the largest proportion (12.0%) within the rarity classes. In contrast, the rarity classes with two rare categories (NRL, NBS, WRS) exhibited lower species numbers, with only one, eight, and two species, respectively. Conversely, the classes with two common categories (NBL, WRL, WBS) included higher numbers of species, totaling 6, 12, and 6 species, respectively. The WBL category, representing the most common species, encompassed 12 species (9.0%).

**Figure 4:**
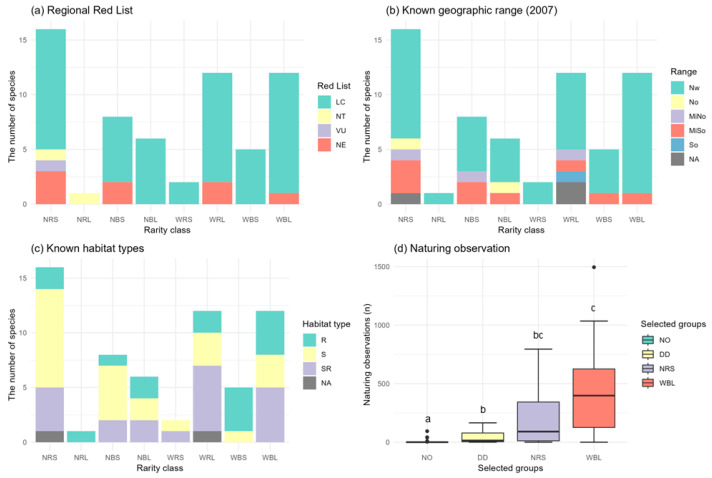
Species count and observation numbers across different criteria. Panel (**a**) displays the number of species within rarity classes as categorized by regional Red List status. Panel (**b**) illustrates the known geographic range of species as of 2007, while panel (**c**) presents the known habitat types. Panel (**d**) depicts the distribution of observation counts on Naturing.net across selected rarity and data status groups, represented by box and whisker plots. The significant difference revealed by Kruskal–Wallis test was indicated by letters (a, b, bc, c) above each box in the panel (**d**). The whiskers extend to 1.5 times the interquartile range (IQR), and outliers are indicated as individual points. For definitions of abbreviations used in the legends, please refer to the Materials and Methods section.

Although the LC species constitute the majority in most rarity classes, only the NRS and NRL classes include species with a higher conservation status according to the current regional Red List (Figure 4a). Notably, three species with wide geographic ranges have not been considered in the current regional Red List assessment. Among the five Odonata species identified in northern or mid-northern regions in 2007 [50], four are generally classified within the narrow geographic range groups (Figure 4b). Species that were commonly observed in southern or mid-southern regions in the past are assigned to various rarity groups. The WBL class consists of species that are frequently observed nationwide. Within the NRS class, species primarily found in lentic environments (S) are predominant, while those associated with R (running water) or SR (both still water and running water) habitat types are relatively more represented in the WBL class (Figure 4c).

The Kruskal–Wallis test revealed a significant difference in the number of observations recorded on Naturing.net among the NO, DD, NRS, and WBL groups (Kruskal–Wallis test, H = 49.6, N = 99, *p* < 0.001) (Figure 4d). Pairwise comparisons using the Wilcoxon rank-sum test with Bonferroni correction indicates that both the NO and DD groups exhibited significantly fewer observations compared to the WBL rarity class (NO vs. WBL: *p* < 0.001; DD vs. WBL: *p* < 0.01). Although the NRS class had a lower mean number of observations compared to the WBL class, the difference was not statistically significant (*p* > 0.05). This lack of significance is likely due to the frequent observations of species such as *Sympetrum pedemontanum*, *Lyriothemis pachygastra*, and *Pantala flavescens*, which had over 500 observations recorded by citizen scientists. The NRS class demonstrated a higher mean number of Naturing.net observations compared to the NO and DD groups; however, only the comparison with the NO group was statistically significant (NRS vs. NO: *p* < 0.001; NRS vs. DD: *p* > 0.05).

Potential explanations for the low data availability of 71 Odonata species categorized as NO or DD in this study were obtained via a review of the literature (Table 5 and Table S1). A common issue was the complete absence of occurrence records or reliable recent observations, affecting 71.4% of species classified as NO and 25.0% of those categorized as DD. Additionally, species with limited and uncommon habitats or low population densities were more prevalent in the DD category (63.9%) compared to the NO category (20.0%). Furthermore, species primarily found in lentic habitats constituted 66.7% of the DD category, whereas they made up only 20.0% of the NO category. Species with relatively narrow geographic ranges were also less frequently recorded, comprising 5.7% of the NO category and 25.0% of the DD category.

## 4. Discussion

### 4.1. Discussion on Occupancy Model Results

This study employed the multispecies occupancy model scheme to estimate occupancy and distribution of Odonata species in South Korea because prior studies demonstrated multispecies occupancy models are effective in handling imperfect detection and occupancy estimation for diverse insect communities [68,69]. The LFMSO models were selected over standard MSO models because they not only account for imperfect detection, as the MSO models do, but also effectively manage residual species correlations among detections [64]. In other words, LFMSO models provide a nuanced understanding of species co-occurrence patterns and achieve a more accurate fit for the data by capturing relationships that are not directly observable from site covariates alone. Interestingly, the general trends of site covariates, including bio1, bio12, lentic and lotic, were largely consistent across both NES and SEAEH data-based LFMSO models, as the species that commonly appeared in both data sources exhibited positive relationships in site covariate estimates (Table S3). However, bio15 (i.e., precipitation seasonality) demonstrated opposite effects on the occurrence probability, even among species commonly found in the Odonata community. This discrepancy in the effects of bio15 between the two models may be linked to differences in the underlying survey methodologies and the resulting species pools. Unlike SEAEH, the NES survey encompasses both representative lentic and lotic environments within the extent of a survey site. Consequently, species that are characteristic of lentic habitats, such as *Anax guttatus* and *Ischnura senegalensis*, are absent from the SEAEH data. Furthermore, the stable vegetation communities along riparian zones of lentic habitats in NES are maintained in areas with lower seasonal precipitation variability [70,71], which could explain why these species exhibit a stronger negative effect of bio15 compared to other species within the NES dataset. In SEAEH, the absence of these lentic habitat specialists, coupled with sampling mainly conducted in areas that are relatively insensitive to seasonality, may result in occupancy modeling estimates being more influenced by the distribution characteristics of the remaining species pool. This, in turn, could account for the inconsistent effects of bio15 observed between the two models.

Positive relationships between bio1 (i.e., annual mean temperature) and occurrence probability were primarily associated with species such as *Paracercion calamorum* and *Orthetrum albistylum*, which are known to inhabit lowland lentic habitats. Conversely, negative relationships with bio1 were linked to species such as *Sieboldius albardae* and *Davidius lunatus*, typically found in mountainous streams [60,61]. Interestingly, species found in southern or mid-southern regions did not consistently exhibit a positive relationship with bio1 when categorized based on latitudinal aspects (Table S1). In arid climates of West Asia and Central Asia, bio12 (i.e., annual precipitation) has been found to positively impact Odonata species richness [72]. Although our study area is not characterized by an overall dry climate, the surveys were conducted during relatively dry seasons (spring and autumn) when intense rainfall does not significantly affect the survival of Odonata nymphs and eggs [73]. The higher annual precipitation in our study area likely ensures more stable freshwater habitats during these sampling seasons, thereby positively influencing the overall occurrence probability of the Odonata community.

### 4.2. The Gap Between the Model Outputs and Existing Data

The occupancy models based on the NES and SEAEH data effectively addressed biased sampling efforts by incorporating survey counts at each site defined by a 1 km grid. However, certain detection variables, such as the effects of survey date and observer bias, could not be applied due to the necessity of spatial and temporal aggregation of the original data. This aggregation was essential to ensure a sufficient number of sites with replicates [74], but it limited the ability to address detection bias arising from open data sources. Additionally, there was a noticeable sampling bias favoring lotic environments, which may have skewed the covariate estimates related to habitat preference, particularly in cases where lentic species were underrepresented. Such biases can adversely affect the model’s ability to accurately predict the occupancy of Odonata species, especially for those with specialized requirements for lotic habitats. While the National Survey on Inland Wetlands (NSIW) was conducted concurrently with the data used in this study [75], occupancy modeling using the NSIW data was not feasible due to the lack of multiple visits or replicates necessary to account for imperfect detection of rare species.

Integrating multiple data sources into a single hierarchical modeling framework can enhance our understanding of species occupancy patterns [37,76,77]. However, this integration requires highly standardized survey methods to ensure data compatibility and reliability. In this study, the spatial and temporal aggregation necessary for standardization limited the ability to capture the full range of detection variability and habitat characteristics at a higher spatial resolution. This underscores the need for survey protocols that balance standardization with flexibility. Even if such integration is achievable, there are technical limitations within the available statistical tools. For instance, the ‘spOccupancy’ package used in this study does not support posterior predictive checks in models that integrate multiple data sources, which are crucial for validating model performance. Therefore, advancements in statistical methodologies are also necessary to ensure more accurate and robust occupancy estimations.

### 4.3. Gap Between Existing Knowledge and a Data-Based Rarity Assessment

Our study highlights both the alignments and discrepancies between the existing ecological knowledge of Odonata species and their data-based rarity status in South Korea. For instance, species such as *Ischnura elegans* and *Aeshna juncea*, which were previously documented in northern regions [61] and are expected to experience range contractions due to climate change, predominantly fell into the narrow geographic range categories in our analysis. Additionally, species classified as NT or VU according to the regional Red List [25] were assigned to high-rarity classes or low-data-availability groups. For example, *Asiagomphus melanopsoides*, *Macromia daimoji*, and *Asiagomphus coreanus*—all categorized as VU on the regional Red List—were included in the NRS or DD categories in our study. Similarly, species categorized as NO were primarily recorded only in North Korea or lacked recent observations in South Korea, further aligning with expectations based on existing knowledge.

Despite these positive alignments with existing knowledge, notable discrepancies were also observed. Many species known to prefer lentic environments were grouped into the DD or NRS categories, suggesting that data collection methods or sampling locations may have introduced biases. For example, lentic Odonata species categorized as DD or NRS, such as *Anax parthenope*, *Anax nigrofasciatus*, *Ceriagrion nipponicum*, and *Sympetrum risi*, have been frequently observed by citizen scientists and are known to be widely distributed [60,61], yet they are assigned as LC species in the current regional Red List criteria [25]. An interesting exception is the case of *Nihonogomphus ruptus*, which is currently assessed as LC on the regional Red List and is known to prefer lotic habitats but was classified as DD in our analysis. This indicates that such species may require re-evaluation through more targeted surveys and comprehensive data collection to determine whether they are genuinely rare or classified as DD due to incomplete data.

### 4.4. Addressing Data Limitations and Improving Survey Strategies for a Reliable Rarity Assessment

One of the primary reasons for the observed gaps between expert knowledge and data-based rarity assessments in this study is the lack of available data that accurately reflects the comprehensive understanding of Odonata species by experts. This issue is particularly relevant for rare species, as the multispecies occupancy modeling framework inherently requires large datasets to establish replicates for reliable occupancy estimates [78,79]. Although nationwide surveys aim for long-term, repeated data collection, they often fall short of achieving high spatial resolution that adequately represents the ecological niches of specific species. This limitation arises primarily from the design of surveys, such as the NES and SEAEH, which were not specifically developed to assess the conservation status of a particular taxon. Instead, these surveys target a broad range of taxa beyond Odonata to identify the current occurrences of various species and assess the ecological health of freshwater ecosystems [7,8]. To enhance survey efficiency, particularly for individual taxa within freshwater ecosystems that share similar niches, future surveys could leverage results from multispecies occupancy models, such as those presented in Figure 3. Identifying areas with potentially high species richness could prioritize these locations for repeated sampling, thereby optimizing data collection efforts and focusing on sites that contribute most to understanding species distribution patterns.

Moreover, improving both the quality and accessibility of ecological data is essential. For instance, some species included in our final model, such as *Enallagma cyathigerum* and *Sympetrum flaveolum*, are reported to have never been found in South Korea. Despite the mandatory involvement of taxonomic experts in the NES and SEAEH programs, previous surveys lacked mechanisms to verify the quality of individual records, such as specimen validation, photographs, or DNA barcoding through open sources. Additionally, accessing data for legally protected species [80], such as *Libellula angelina* and *Nannophya koreana*, proved challenging due to restrictions imposed by data providers. This issue was particularly evident for *Libellula angelina*, which was classified as DD in our study despite numerous observations (*n* > 100) by citizen scientists (Table S1). While policies restricting access to data on species sensitive to habitat destruction are understandable, there is a compelling need for balanced approaches to data sharing. Adopting models similar to the National Framework for the Sharing of Restricted Access Species Data in Australia (RASD) [81], where sensitive information is made available to verified researchers and institutional stakeholders for legitimate research purposes, could provide a starting point for more neutral and accurate assessments of rare and endangered species. Ensuring data accessibility and traceability would allow ecologists and conservation scientists to conduct more robust evaluations of species’ conservation statuses.

## 5. Conclusions

This study underscores the need to enhance both survey methodologies and data accessibility to achieve more accurate, data-driven rarity assessments. The application of multispecies occupancy models has demonstrated their potential to provide valuable insights into species occurrence patterns and reveal limitations inherent in current data collection practices. However, it is important to acknowledge that multispecies occupancy models have inherent limitations. Species distributions are often influenced by a range of environmental and ecological factors, or their combinations, that the models may not fully capture. Future studies should account for these complex interactions to better understand actual species occupancy.

The observed discrepancies between data-driven assessments and expert knowledge highlight the challenges associated with biased sampling efforts, limited data availability, and the need for more standardized survey schemes that can capture the complexities of species occurrences. Furthermore, our findings suggest that more refined data-sharing policies, particularly for sensitive species, and advancements in statistical tools are crucial for addressing existing data limitations and biases. Future efforts should also focus on integrating data from multiple sources within a unified modeling framework that balances the need for standardization with computational efficiency. By addressing these gaps and incorporating robust, validated data into rarity assessments, we can significantly enhance our capacity to develop reliable biodiversity conservation strategies and inform policy decisions.

## Figures and Tables

**Table 1:** Environmental variables collected for occupancy models and the Rabinowitz framework.

Types	Description	Unit	Data Source
Topographic	Height above sea level	Meter	ALOS DSM: Global 30m v3.2 (https://developers.google.com/earth-engine/datasets (Accessed on 10 April 2024))
Land-cover	Forest, agriculture, urban, lentic, and lotic areas	Square meter	Land-cover maps (https://egis.me.go.kr/ (Accessed on 10 April 2024))
Bioclimatic variables	Total of 19 variables (bio1–bio19) calculated from monthly temperature and rainfall values	°C, millimeter, and ratio	WorldClim (https://www.worldclim.org (Accessed on 10 April 2024))

**Table 2:** Summary of site covariates of the LFMSO models from NES and SEAEH data.

Covariates	Estimates (Mean ± SD)		R-Hat Convergence Diagnostic		Effective Sample Size	
	NES	SEAEH	NES	SEAEH	NES	SEAEH
(Intercept)	0.10 ± 0.40	−1.64 ± 0.24	1.06	1.03	131	466
bio1	0.44 ± 0.21	0.54 ± 0.15	1.03	1.00	1080	1409
bio12	−0.57 ± 0.21	−0.40 ± 0.09	1.02	1.00	779	1405
bio15	−0.29 ± 0.17	0.28 ± 0.09	1.02	1.00	452	1765
Lentic	−0.10 ± 0.17	−0.10 ± 0.07	1.01	1.01	1489	1152
Lotic	0.10 ± 0.15	0.09 ± 0.05	1.00	1.00	1057	1567

**Table 3:** Summary of detection covariates of the LFMSO from NES and SEAEH data.

Covariates	Estimates (Mean ± SD)		R-Hat Convergence Diagnostic		Effective Sample Size	
	NES	SEAEH	NES	SEAEH	NES	SEAEH
(Intercept)	−2.59 ± 0.26	−3.91 ± 0.21	1.02	1.03	410	435
Visit number	0.21 ± 0.09	0.15 ± 0.02	1.000	1.00	2803	2803

**Table 4:** Summary of data status and rarity classes of Odonata species by using the Rabinowitz framework based on the LFMSO results.

Data Availability for Modeling	Range	Habitat Specificity	Local Population	Rarity Class	All Species	
					*n*	%
Not observed	-	-	-	-	35	26.3
Data deficient					36	27.1
Available	Narrow	Restricted	Small	NRS (rare)	16	12.0
			Large	NRL	1	0.8
		Broad	Small	NBS	8	6.0
			Large	NBL	6	4.5
	Wide	Restricted	Small	WRS	2	1.5
			Large	WRL	12	9.0
		Broad	Small	WBS	5	3.8
			Large	WBL (common)	12	9.0

**Table 5:** Summary of primary reasons for the low data availability of Odonata species.

Reason for Low Data Availability	Data Status	All Species	
		*n*	%
No occurrence records or reliable recent observations in South Korea	NO	25	71.4
	DD	9	25.0
Limited and uncommon habitats or low population density	NO	7	20.0
	DD	23	63.9
Primarily found in lentic habitats	NO	7	20.0
	DD	24	66.7
Relatively narrow geographic range	NO	2	5.7
	DD	9	25.0

## Data Availability

The data that underpin the findings in this study are provided in the Supplementary Materials.

## Supplementary Materials

The following supporting information can be downloaded at: https://www.mdpi.com/article/10.3390/insects15110887/s1, Table S1: Comprehensive overview of Odonata species in South Korea: integrating existing knowledge, rarity assessment, and discussion on low data availability [82,83,84,85,86,87], Table S2: Estimate values of site and detection covariates for Odonata species using the LFMSO models from NES and SEAEH data, Table S3: Correlation analysis of mean site covariate estimates of the LFMSO models for commonly observed Odonata species from NES and SEAEH data.

## References

[1] Abbott J.C., Bota-Sierra C.A., Guralnick R., Kalkman V., González-Soriano E., Novelo-Gutiérrez R., Bybee S., Ware J., Belitz M.W. (2022). Diversity of Nearctic Dragonflies and Damselflies (Odonata). Diversity.

[2] Arnaud S., Kari K., Lenin C., André M., Olga P., Justin P., David B., Nedim T., Robby S., Cordoba-Auilar A., Beatty C., Bried J. (2022). Odonata Trophic Ecology: From Hunting Behavior to Cross-Ecosystem Impact. Dragonflies and Damselflies: Model Organisms for Ecological and Evolutionary Research.

[3] Clarke A., Prince P.A., Clarke R. (1996). The Energy Dontent of Dragonflies (Odonata) in Relation to Predation by Falcons. Bird Study.

[4] Miguel T.B., Oliveira-Junior J.M.B., Ligeiro R., Juen L. (2017). Odonata (Insecta) as a Tool for the Biomonitoring of Environmental Quality. Ecol. Indic..

[5] Hassall C. (2015). Odonata as Candidate Macroecological Barometers for Global Climate Change. Freshw. Sci..

[6] Souza N.F., Leal J.S., Tourinho L., Farjalla V.F., Rocha D.S.B., Vale M.M. (2024). Bioindicator Aquatic Insects at Risk from Climate Change in a Biodiversity Hotspot. Sci. Total Environ..

[7] Gómez-Tolosa M., Rivera-Velázquez G., Rioja-Paradela T.M., Mendoza-Cuenca L.F., Tejeda-Cruz C., López S. (2021). The Use of Odonata Species for Environmental Assessment: A Meta-Analysis for the Neotropical Region. Environ. Sci. Pollut. Res..

[8] Datto-Liberato F.H., Felipe H., Lopez V.M., Quinaia T., Farias do Valle Junior R., Samways M.J., Juen L., Valera C., Guillermo-Ferreira R. (2024). Total Environment Sentinels: Dragonflies as Ambivalent/Amphibiotic Bioindicators of Damage to Soil and Freshwater. Sci. Total Environ..

[9] Rocha-Ortega M., Rodríguez P., Bried J., Abbott J., Córdoba-Aguilar A. (2020). Why Do Bugs Perish? Range Size and Local Vulnerability Traits as Surrogates of Odonata Extinction Risk. Proc. R. Soc. B Biol. Sci..

[10] Tang D.H.Y., Visconti P. (2021). Biases of Odonata in Habitats Directive: Trends, Trend Drivers, and Conservation Status of Europen Threatened Odonata. Insect Conserv. Divers..

[11] White E.L., Hunt P.D., Schlesinger M.D., Corser J.D., De Maynadier P.G. (2015). Prioritizing Odonata for Conservation Action in the Northeastern USA. Freshw. Sci..

[12] Elo M., Penttinen J., Kotiaho J.S. (2015). The Effect of Peatland Drainage and Restoration on Odonata Species Richness and Abundance. BMC Ecol..

[13] Goodman A.M., Kass J.M., Ware J. (2023). Dynamic Distribution Modelling of the Swamp Tigertail Dragonfly *Synthemis eustalacta* (Odonata: Anisoptera: Synthemistidae) over a 20-Year Bushfire Regime. Ecol. Entomol..

[14] Kašák J., Holuša O., Mazalová M. (2023). Artificial habitat—A Chance for Survival of a Rare Montane Dragonfly (Odonata): Case Study on an Alpine Emerald (*Somatochlora alpestris*). J. Insect Conserv..

[15] Pires M., Martins F., del Palacio A., Muzón J., Vareira L., Juen L., Périco E. (2024). Assessing the Spatial Knowledge Gaps of Odonata Diversity and Conservation in the South American Pampa. Aquat. Conserv..

[16] Zhao Z., Feng X., Zhang Y., Wang Y., Zhou Z. (2023). Species Diversity, Hotspot Congruence, and Conservation of North American Damselflies (Odonata: Zygoptera). Front. Ecol. Evol..

[17] Beaujour P.M., Loranger-Merciris G., Cézilly F. (2024). Sites and Species Contribution to the β-Diversity of Odonata Assemblages in Haiti: Implications for Conservation. Glob. Ecol. Conserv..

[18] Kim H.G., Jang R.H., Kim S., Tho J.H., Jung J.W., Cheong S., Yoon Y.J. (2022). Developing Habitat Suitability Index for Habitat Evaluation of *Nannophya koreana* Bae (Odonata: Libellulidae). J. Ecol. Environ..

[19] Hong J., Kwon S.J., Lee C.S., Choi J.Y., Cho K., Kim H.G. (2023). Potential Distribution of the Critically Endangered Dragonfly *Libellula angelina* (Odonata: Libellulidae) under Shared Socio-Economic Pathways. Entomol. Res..

[20] Shin S., Jung K.S., Kang H.G., Dang J.H., Kang D., Han J.E., Kim J.H. (2021). Northward Expansion Trends and Future Potential Distribution of a Dragonfly *Ischnura senegalensis* Rambur under Climate Change Using Citizen Science Data in South Korea. J. Ecol. Environ..

[21] Choi J.Y., Kim S.K., Kim J.C., Kwon S.J. (2020). Habitat Preferences and Trophic Position of *Brachydiplax chalybea flavovittata* Ris, 1911 (Insecta: Odonata) Larvae in Youngsan River Wetlands of South Korea. Insects.

[22] Hwang J.H., Kim E., Choi E.Y., Choi J.B., Park J.K. (2020). Insect Diversity in Gonggeom-Ji, the First Protected Paddy Field Wetland in Korea. Entomol. Res..

[23] Lee S.D., Bae S.H., Lee G.G. (2020). Understanding the Impact of Environmental Changes on the Number of Species and Populations of Odonata after Creating a Constructed Wetland. Korean J. Environ. Ecol..

[24] Shin I.C., Kim M.H., Eo J. (2022). Analysis of Community Stability and Characteristics of Macroinvertebrates in Paddy Fields by Cultivation Method. Ecol. Resil. Infrastruct..

[25] National Institute of Biological Resources (2023). Red Data Book of Republic of Korea Volume 9. Insect III.

[26] Cuevas-Yáñez K., Rivas M., Muñoz J., Córdoba-Aguilar A. (2015). Conservation Status Assessment of *Paraphlebia* Damselflies in Mexico. Insect Conserv. Divers..

[27] Collins S.D., McIntyre N.E. (2015). Modeling the Distribution of Odonates: A Review. Freshw. Sci..

[28] Boys W.A., Siepielski A.M., Smith B.D., Patten M.A., Bried J.T. (2021). Predicting the Distributions of Regional Endemic Dragonflies Using a Combined Model Approach. Insect Conserv. Divers..

[29] Hassall C. (2012). Predicting the Distributions of Under-Recorded Odonata Using Species Distribution Models. Insect Conserv. Divers..

[30] Domisch S., Jähnig S.C., Haase P. (2011). Climate-Change Winners and Losers: Stream Macroinvertebrates of a Submontane Region in Central Europe. Freshw. Biol..

[31] Mackenzie D.I., Royle J.A. (2005). Designing Occupancy Studies: General Advice and Allocating Survey Effort. J. Appl. Ecol..

[32] Veloz S.D. (2009). Spatially Autocorrelated Sampling Falsely Inflates Measures of Accuracy for Presence-Only Niche Models. J. Biogeogr..

[33] Hayward M.W., Child M.F., Kerley G.I.H., Lindsey P.A., Somers M.J., Burns B. (2015). Ambiguity in Guideline Definitions Introduces Assessor Bias and Influences Consistency in IUCN Red List Status Assessments. Front. Ecol. Evol..

[34] Bachman S.P., Field R., Reader T., Raimondo D., Donaldson J., Schatz G.E., Lughadha E.N. (2019). Progress, Challenges and Opportunities for Red Listing. Biol. Conserv..

[35] Breiner F.T., Guisan A., Bergamini A., Nobis M.P. (2015). Overcoming Limitations of Modelling Rare Species by Using Ensembles of Small Models. Methods Ecol. Evol..

[36] van Proosdij A.S.J., Sosef M.S.M., Wieringa J.J., Raes N. (2016). Minimum Required Number of Specimen Records to Develop Accurate Species Distribution Models. Ecography.

[37] Belmont J., Miller C., Scott M., Wilkie C. (2022). A New Statistical Approach for Identifying Rare Species under Imperfect Detection. Divers. Distrib..

[38] Doser J.W., Leuenberger W., Sillett T.S., Hallworth M.T., Zipkin E.F. (2022). Integrated Community Occupancy Models: A Framework to Assess Occurrence and Biodiversity Dynamics Using Multiple Data Sources. Methods Ecol. Evol..

[39] Roberts D.L., Hinsley A., Fiennes S., Veríssimo D. (2023). Understanding the Drivers of Expert Opinion When Classifying Species as Extinct. Conserv. Biol..

[40] Samways M.J., Grant P.B.C. (2007). Honing Red List Assessments of Lesser-Known Taxa in Biodiversity Hotspots. Biodivers. Conserv..

[41] Lahti K., Schulman L., Piirainen E., Riihikoski V.-M., Juslén A. (2019). In ‘As Open as Possible, as Closed as Necessary’—Managing Legal and Owner-Defined Restrictions to Openness of Biodiversity Data. BISS.

[42] Higgs P., Slatyer C. (2023). Restricted Access Species Data Systems: A Starting Point. BISS.

[43] Choe H., Thorne J.H., Hijmans R., Seo C. (2019). Integrating the Rabinowitz Rarity Framework with a National Plant Inventory in South Korea. Ecol. Evol..

[44] Korea Meteorological Administration The Climatic Characteristics of South Korea. https://www.weather.go.kr/w/climate/statistics/korea-char.do.

[45] Water Environment Information System Biomonitoring Survey: Macrobenthic Invertebrate. http://water.nier.go.kr/.

[46] EcoBank Open API Data: The Point-Based Macrobenthic Invertebrate Data from National Ecosystem Survey. https://nie-ecobank.kr.

[47] Engelhardt E.K., Biber M.F., Dolek M., Fartmann T., Hochkirch A., Leidinger J., Löffler F., Pinkert S., Poniatowski D., Voith J. (2022). Consistent Signals of a Warming Climate in Occupancy Changes of Three Insect Taxa over 40 Years in Central Europe. Glob. Change Biol..

[48] Cerini F., Vignoli L., Blust M., Strona G. (2023). Functional Traits Predict Species Co-Occurrence Patterns in a North American Odonata Metacommunity. Ecosphere.

[49] O’Neill D., Shaffrey L., Neumann J., Cheffings C., Norris K., Pettorelli N. (2024). Investigating Odonates’ Response to Climate Change in Great Britain: A Tale of Two Strategies. Divers. Distrib..

[50] Pinkert S., Zeuss D., Dijkstra K.D.B., Kipping J., Clausnitzer V., Brunzel S., Brandl R. (2020). Climate–Diversity Relationships Underlying Cross-Taxon Diversity of the African Fauna and Their Implications for Conservation. Divers. Distrib..

[51] Deacon C., Samways M.J., Pryke J.S. (2020). Determining Drivers of Dragonfly Diversity Patterns and the Implications for Conservation in South Africa. Biol. Conserv..

[52] Acquah-Lamptey D., Brändle M., Brandl R., Pinkert S. (2020). Temperature-Driven Color Lightness and Body Size Variation Scale to Local Assemblages of European Odonata but Are Modified by Propensity for Dispersal. Ecol. Evol..

[53] Kalkman V.J., Boudot J.P., Futahashi R., Abbott J.C., Bota-Sierra C.A., Guralnick R., Bybee S.M., Ware J., Belitz M.W. (2022). Diversity of Palaearctic Dragonflies and Damselflies (Odonata). Diversity.

[54] Li F., Park Y.S. (2020). Habitat Availability and Environmental Preference Drive Species Range Shifts in Concordance with Climate Change. Divers. Distrib..

[55] Gil-Tapetado D., López-Collar D., Gómez J.F., Mañani-Pérez J., Cabrero-Sañudo F.J., Muñoz J. (2023). Climate Change as a Driver of Insect Invasions: Dispersal Patterns of a Dragonfly Species Colonizing a New Region. PLoS ONE.

[56] Saxton N.A., Paxman E.M., Dean A.M., Jensen C.R., Powell G.S., Bybee S.M. (2021). Factors Influencing the Distribution of Endemic Damselflies in Vanuatu. Insects.

[57] Viza A., Garcia-Raventós A., Riera J.L., Maynou X., Martín R., Prunier F., El Haissoufi M., Múrria C. (2023). Species-Specific Functional Traits Rather than Phylogenetic Relatedness Better Predict Future Range-Shift Responses of Odonates. Insect Conserv. Divers..

[58] Cancellario T., Miranda R., Baquero E., Fontaneto D., Martínez A., Mammola S. (2022). Climate Change Will Redefine Taxonomic, Functional, and Phylogenetic Diversity of Odonata in Space and Time. Npj Biodivers..

[59] ESRI (2023). ArcGIS Pro.

[60] Cho S., Seo C., Kim D. (2021). Korean Odonata Adult and Larva.

[61] Jung K.S. (2007). Odonata Larvae of Korea.

[62] Doser J.W., Finley A.O., Kéry M., Zipkin E.F. (2022). spOccupancy: An R Package for Single-Species, Multi-Species, and Integrated Spatial Occupancy Models. Methods Ecol. Evol..

[63] Zipkin E.F., Doser J.W., Davis C.L., Leuenberger W., Ayebare S., Davis K.L. (2023). Integrated Community Models: A Framework Combining Multispecies Data Sources to Estimate the Status, Trends and Dynamics of Biodiversity. J. Anim. Ecol..

[64] Doser J.W., Finley A.O., Banerjee S. (2023). Joint Species Distribution Models with Imperfect Detection for High-Dimensional Spatial Data. Ecology.

[65] R Core Team (2024). R: A Language and Environment for Statistical Computing.

[66] Shannon C.E. (1948). A Mathematical Theory of Communication. Bell Syst. Tech. J..

[67] Maechler M., Rousseeuw P., Struyf A., Hubert M., Hornik K. R Package, Version 2.1.6; cluster: Cluster Analysis Basics and Extensions: 2023. https://CRAN.R-project.org/package=cluster.

[68] Boone M.L., Evans E., Arnold T., Cariveau D.P. (2023). Increasing sampling efficiency of Bombus communities with rare and endangered species by optimizing detection probabilities: A multi-species occupancy modelling approach using roadsides as a case study. Biol. Conserv..

[69] Mourguiart B., Couturier T., Braud Y., Mansons J., Combrisson D., Besnard A. (2021). Multi-species occupancy models: An effective and flexible framework for studies of insect communities. Ecol. Entomol..

[70] Butterfield B.J., Palmquist E.C., Yackulic C.B. (2023). The Hydroclimate Niche: A Tool for Predicting and Managing Riparian Plant Community Responses to Streamflow Seasonality. River. Res. Appl..

[71] Portela A.P., Durance I., Vieira C., Honrado J. (2023). Environmental Filtering and Environmental Stress Shape Regional Patterns of Riparian Community Assembly and Functional Diversity. Freshw. Biol..

[72] Cadena J.T., Boudot J.P., Kalkman V.J., Marshall L. (2023). Impacts of Climate Change on Dragonflies and Damselflies in West and Central Asia. Divers. Distrib..

[73] Thompson D.J. (1990). The Effects of Survival and Weather on Lifetime Egg Production in a Model Damselfly. Ecol. Entomol..

[74] Kéry M., Gardner B., Monnerat C. (2010). Predicting species distributions from checklist data using site-occupancy models. J. Biogeogr..

[75] EcoBank DOI Dataset: The Biota Data of National Survey on the Inland Wetlands. 2016–2021. https://www.nie-ecobank.kr/rdm/rsrchdoi/selectRsrchDtaDtlVw.do?rsrchDtaId=RSD_0000000000012670.

[76] Emmet R.L., Benson T.J., Allen M.L., Stodola K.W. (2023). Integrating Multiple Data Sources Improves Prediction and Inference for Upland Game Bird Occupancy Models. Ornithol. Appl..

[77] Pacifici K., Reich B.J., Miller D.A.W., Gardner B., Stauffer G., Singh S., McKerrow A., Collazo J.A. (2017). Integrating Multiple Data Sources in Species Distribution Modeling: A Framework for Data Fusion. Ecology.

[78] Santos R.A.L., Mota-Ferreira M., Aguiar L.M.S., Ascensão F. (2018). Predicting Wildlife Road-Crossing Probability from Roadkill Data Using Occupancy-Detection Models. Sci. Total Environ..

[79] Specht H.M., Reich H.T., Iannarilli F., Edwards M.R., Stapleton S.P., Weegman M.D., Johnson M.K., Yohannes B.J., Arnold T.W. (2017). Occupancy Surveys with Conditional Replicates: An Alternative Sampling Design for Rare Species. Methods Ecol. Evol..

[80] National Institute of Ecology, The Endangered Species List of South Korea. https://www.nie.re.kr/nie/pgm/edSpecies/edSpeciesList.do?menuNo=200127.

[81] Atlas of Living Australia (2023). National Framework for the Sharing of Restricted Access Species Data in Australia 2023.

[82] Dow R.A. *Pseudagrion pilidorsum*, The IUCN Red List of Threatened Species. e.T139350609A139403898. 2021. https://www.iucnredlist.org/species/139350609/139403898.

[83] Subramanian K.A. *Rhyothemis variegata*. The IUCN Red List of Threatened Species. e.T167133A83384189. 2020. https://www.iucnredlist.org/species/167133/83384189.

[84] Subramanian K.A. *Neurothemis fluctuans*. The IUCN Red List of Threatened Species. e.T167488A83383506. 2020. https://www.iucnredlist.org/species/167488/83383506.

[85] Dow R.A. *Gynacantha basiguttata* (Amended Version of 2011 Assessment). The IUCN Red List of Threatened Species e.T167287A176402482. 2020. https://www.iucnredlist.org/species/167287/176402482.

[86] Dow R.A. *Orthetrum luzonicum*. The IUCN Red List of Threatened Species. e.T167309A6326889. 2010. https://www.iucnredlist.org/species/167309/6326889.

[87] Jung K.S. (2012). The Drgonflies and Damselflies in Korea.

